# Adipsic hypernatremia associated with hypothalamic dysfunction and sleep disorders

**DOI:** 10.1210/jcemcr/luag173

**Published:** 2026-07-02

**Authors:** Mahmoud Draidi, Mohamed Ataelmanan, Moaz O Moursi, Anas Aburamadan, Muhammad Naeem

**Affiliations:** Department of Internal Medicine, Hamad Medical Corporation, PO Box 3050, Doha, Qatar; Department of Internal Medicine, Hamad Medical Corporation, PO Box 3050, Doha, Qatar; Department of Internal Medicine, Hamad Medical Corporation, PO Box 3050, Doha, Qatar; Department of Internal Medicine, Hamad Medical Corporation, PO Box 3050, Doha, Qatar; Department of Internal Medicine, Hamad Medical Corporation, PO Box 3050, Doha, Qatar

**Keywords:** adipsic hypernatremia, hypothalamic dysfunction, hypogonadotropic hypogonadism, hyperprolactinemia, narcolepsy with cataplexy, obstructive sleep apnea

## Abstract

Adipsic hypernatremia is a rare hypothalamic disorder characterized by impaired thirst despite preserved renal concentrating ability and may coexist with multisystem neuroendocrine and sleep disturbances. We report a 20-year-old male with beta-thalassemia minor presenting with severe hypernatremia with serum sodium of 170 mEq/L (SI: 170 mmol/L) (reference range 133-146 mEq/L [SI: 133-146 mmol/L]), absent thirst, weight loss, fatigue, hypogonadotropic hypogonadism, and hyperprolactinemia, along with excessive daytime sleepiness, hypnagogic hallucinations, and emotion-triggered cataplexy. Laboratory evaluation confirmed preserved renal concentrating ability, and pituitary magnetic resonance imaging was normal. Findings were consistent with adipsic hypernatremia due to functional hypothalamic dysfunction, with coexisting severe obstructive sleep apnea confirmed on polysomnography (apnea–hypopnea index [AHI] 35.9 events/hour) and presumed narcolepsy with cataplexy. Management included structured fluid intake, testosterone replacement, cabergoline, wake-promoting agents (modafinil, methylphenidate), and continuous positive airway pressure therapy. This case underscores the complex interplay between hypothalamic dysfunction, endocrine abnormalities, and sleep disorders, highlighting the importance of early recognition and structured management to prevent recurrent hypernatremia and optimize clinical outcomes.

## Introduction

Maintenance of plasma osmolality relies on the coordinated activity of hypothalamic osmoreceptors, thirst perception, and arginine vasopressin (AVP) secretion. Even minor increases in plasma osmolality trigger AVP release and a conscious drive to drink, ensuring tight regulation of serum sodium levels. Disruption of hypothalamic thirst mechanisms can result in adipsic hypernatremia, a rare condition characterized by impaired thirst despite preserved renal concentrating ability [1]. Adipsic hypernatremia may occur without structural hypothalamic lesions or polyuria and is often associated with broader hypothalamic dysfunction, including dysregulation of pituitary hormones and sleep-wake disturbances. Recognition of adipsic hypernatremia is clinically important to prevent recurrent hypernatremic episodes and guide management through structured fluid intake and close biochemical monitoring rather than pharmacologic therapy alone [2].

Sleep apnea and narcolepsy type 1 (NT1) frequently coexist, reflecting overlapping hypothalamic pathology, particularly involving orexin (hypocretin) pathways, which play a central role in regulating wakefulness, arousal, and respiratory stability [3]. Sleep apnea contributes to central neuroendocrine stress through intermittent hypoxia, sleep fragmentation, and sympathetic activation, which can disrupt hypothalamic function, impair osmoreceptor activity, and exacerbate adipsic hypernatremia [4]. Understanding the interplay between hypothalamic injury, sleep-disordered breathing, and dysregulated water homeostasis provides a unifying framework for interpreting cases in which patients present with hypernatremia, absent thirst, and hypothalamic dysfunction despite normal structural imaging [1-4].

## Case presentation

A 20-year-old male with beta-thalassemia minor and no other chronic illnesses was referred to the emergency department for critically elevated serum sodium. He had been followed for one year for unintentional weight loss and fatigue. Routine labs prior to a general medicine clinic appointment revealed severe hypernatremia (170 mmol/L). Review of prior labs showed progressive hypernatremia over 12 to 18 months, rising from 156 to 170 mmol/L.

He reported chronic sleep-related symptoms predating the detection of hypernatremia, including excessive sleep duration (>10 hours per night compared with 6-7 hours previously), non-restorative sleep, excessive daytime sleepiness (EDS), fatigue, and cognitive slowing. He also experienced uncontrollable sleep episodes, including one minor motor vehicle accident, frequent hypnagogic hallucinations, and episodes of forceful laughter-triggered bilateral eyelid drooping progressing to complete ptosis.

He denied polyuria; instead, he noted gradual reduction in urine output over 6 months, with darker, more concentrated urine. He reported absent thirst, forgetting to drink unless reminded, and denied gastrointestinal fluid loss, orthostatic symptoms, dry mouth, or dry eyes.

He experienced unintentional weight loss of ∼20 kg, from 95 to 75 kg, stabilizing at 70 kg. Appetite remained poor, with marked fatigue. Sexual history revealed reduced libido and absent morning erections over the past year, without testicular pain, galactorrhea, gynecomastia, changes in body hair, voice changes, or symptoms suggestive of thyroid dysfunction such as heat or cold intolerance, weight change, or palpitations.

History was negative for central nervous system disease, head trauma, neurosurgery, or cranial irradiation. He is a university student, single, and living independently and denies tobacco, alcohol, or recreational drug use. Family history was noncontributory for endocrine, neurological, or sleep disorders.

On examination, the patient was alert and oriented, with a weight of 71 kg, height 178 cm (body mass index [BMI] 22.4 kg/m^2^), and blood pressure of 96/49 mmHg. He appeared clinically dehydrated, with dry mucous membranes and reduced skin turgor, without orthostatic hypotension or peripheral edema. Neurological examination was normal, with intact cranial nerves and no focal deficits. Endocrine examination showed normal secondary sexual characteristics without gynecomastia or features of thyroid dysfunction. Visual fields were grossly intact.

## Diagnostic assessment

**Figure 1:**
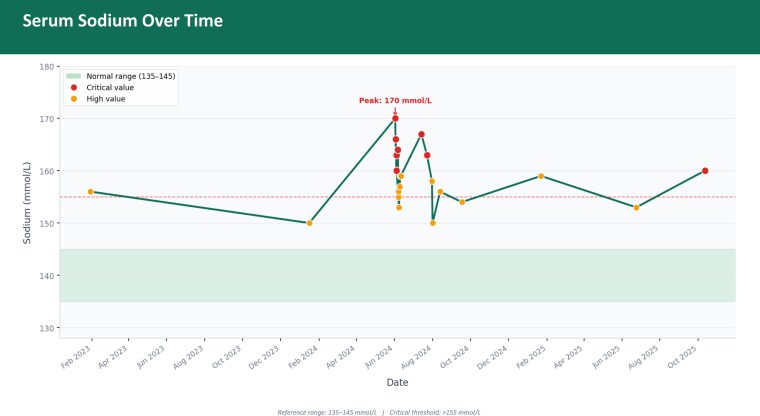
Trend of serum sodium concentration over time. The y-axis represents serum sodium (mmol/L), and the x-axis represents time (date).

Review of prior laboratory data demonstrated normal serum sodium in 2017, followed by progressive hypernatremia from January 2023 (156 mmol/L) to a peak of 170 mmol/L in June 2024 (Fig. 1). Renal function was preserved: creatinine 1.06 mg/dL (SI: 94 µmol/L) (reference range 0.70-1.20 mg/dL [SI: 62-106 µmol/L]), serum urea 64.9 mg/dL (SI: 10.8 mmol/L) (reference range 15.0-46.8 mg/dL [SI: 2.5-7.8 mmol/L]), estimated glomerular filtration rate (eGFR) > 60 mL/min/1.73 m^2^ (reference range > 60 mL/min/1.73 m^2^), and serum potassium, calcium, liver enzymes, albumin, and total protein were normal. Serum bicarbonate ranged 28 to 32 mEq/L (SI: 28-32 mmol/L) (reference range 22-26 mEq/L [SI: 22-26 mmol/L]) (Table 1).

**Table 1:** The extended serum chemistry, complete blood count, lipid panel, iron indices, and hemoglobin electrophoresis results of the patient

Test	Result (conventional units)	Result (SI units)	Reference range
Sodium	170 mEq/L	170 mmol/L	133-146 mEq/L (SI: 133-146 mmol/L)
Potassium	3.8 mEq/L	3.8 mmol/L	3.5-5.3 mEq/L (SI: 3.5-5.3 mmol/L)
Chloride	124 mEq/L	124 mmol/L	95-108 mEq/L (SI: 95-108 mmol/L)
Bicarbonate	32 mEq/L	32 mmol/L	22-26 mEq/L (SI: 22-26 mmol/L)
Urea	64.9 mg/dL	10.8 mmol/L	15.0-46.8 mg/dL (SI: 2.5-7.8 mmol/L)
Creatinine	1.06 mg/dL	94 µmol/L	0.70-1.20 mg/dL (SI: 62-106 µmol/L)
Estimated glomerular filtration rate (eGFR)	>60 mL/min/1.73 m^2^	>60 mL/min/1.73 m^2^	>60 mL/min/1.73 m^2^
Serum osmolality	351 mOsm/kg	351 mOsm/kg	275-295 mOsm/kg
Calcium (adjusted)	9.84 mg/dL	2.46 mmol/L	8.8-10.4 mg/dL (SI: 2.20-2.60 mmol/L)
Phosphorus	2.76 mg/dL	0.89 mmol/L	2.48-4.65 mg/dL (SI: 0.80-1.50 mmol/L)
Magnesium	1.77 mg/dL	0.73 mmol/L	1.70-2.43 mg/dL (SI: 0.70-1.00 mmol/L)
Uric acid	7.70 mg/dL	458 µmol/L	3.4-7.0 mg/dL (SI: 202-416 µmol/L)
Iron	84 µg/dL	15 µmol/L	34-196 µg/dL (SI: 6-35 µmol/L)
Total iron-binding capacity (TIBC)	279 µg/dL	50 µmol/L	251-447 µg/dL (SI: 45-80 µmol/L)
Transferrin	200 mg/dL	2.0 g/L	200-360 mg/dL (SI: 2.0-3.6 g/L)
Iron saturation	30%	30%	15-45%
Ferritin	260 ng/mL	260 µg/L	38-270 ng/mL (SI: 38-270 µg/L)
Total protein	7.4 g/dL	74 g/L	6.0-8.0 g/dL (SI: 60-80 g/L)
Albumin	3.9 g/dL	39 g/L	3.5-5.0 g/dL (SI: 35-50 g/L)
Bilirubin (total)	0.18 mg/dL	3 µmol/L	0-1.2 mg/dL (SI: 0-21 µmol/L)
Alkaline phosphatase (ALP)	70 U/L	70 U/L	40-129 U/L
Alanine aminotransferase (ALT)	20 U/L	20 U/L	0-41 U/L
Aspartate aminotransferase (AST)	20 U/L	20 U/L	0-40 U/L
Hemoglobin A1c (HbA1c)	5.4%	36 mmol/mol	—
Cholesterol	136 mg/dL	3.52 mmol/L	—
Triglycerides	146 mg/dL	1.65 mmol/L	—
High-density lipoprotein (HDL)	28 mg/dL	0.72 mmol/L	—
Low-density lipoprotein (LDL), calculated	80 mg/dL	2.06 mmol/L	—
White blood cell count (WBC)	6.8 × 10^3^/µL	6.8 × 10^9^/L	4.0-10.0 × 10^3^/µL
Red blood cell count (RBC)	6.5 × 10^6^/µL	6.5 × 10^12^/L	4.5-5.5 × 10^6^/µL
Hemoglobin	11.5 g/dL	115 g/L	13.0-17.0 g/dL
Hematocrit	39.9%	0.399 L/L	40.0-50.0%
Mean corpuscular volume (MCV)	61.7 fL	61.7 fL	83.0-101.0 fL
Mean corpuscular hemoglobin (MCH)	17.8 pg	17.8 pg	27.0-32.0 pg
Mean corpuscular hemoglobin concentration (MCHC)	28.8 g/dL	288 g/L	31.5-34.5 g/dL
Red cell distribution width (RDW)	20.6%	20.6%	11.6-14.0%
Platelets	157 × 10^3^/µL	157 × 10^9^/L	150-410 × 10^3^/µL
Hemoglobin A	94.1%	94.1%	95.8-98.0%
Hemoglobin A2	5.5%	5.5%	2.0-3.3%
Hemoglobin F	0.4%	0.4%	0.0-0.9%

Abbreviations: eGFR, estimated glomerular filtration rate; TIBC, total iron-binding capacity; ALP, alkaline phosphatase; ALT, alanine aminotransferase; AST, aspartate aminotransferase; HbA1c, hemoglobin A1c; HDL, high-density lipoprotein; LDL, low-density lipoprotein; WBC, white blood cell count; RBC, red blood cell count; MCV, mean corpuscular volume; MCH, mean corpuscular hemoglobin; MCHC, mean corpuscular hemoglobin concentration; RDW, red cell distribution width.

Endocrine evaluation revealed hypogonadotropic hypogonadism with low total testosterone at 270 ng/dL (SI: 9.38 nmol/L) (reference range 300-1077 ng/dL [SI: 10.4-37.4 nmol/L]) and low follicle-stimulating hormone (FSH) (1.4 IU/L) (SI: 1.4 IU/L) (reference range 1.5-12.4 IU/L), with normal luteinizing hormone (LH) at 7.0 IU/L (SI: 7.0 IU/L) (reference range 1.7-8.6 IU/L). Over a 2-year follow-up period, repeated hormonal assessments showed persistently low FSH with normal LH and low to low-normal testosterone levels. Thyroid function and insulin-like growth factor 1 (IGF-1) were normal. Prolactin was elevated at 101.2 ng/mL (SI: 2146 mIU/L) (reference range 4.0-15.2 ng/mL [SI: 85-323 mIU/L]). Morning cortisol was initially low at 3.8 µg/dL (SI: 105 nmol/L) (reference range 5.0-25.0 µg/dL [SI: 138-689 nmol/L]) with normal adrenocorticotrophic hormone (ACTH) level at 15.7 pg/mL (SI: 15.7 pg/mL) (reference range 7.2-63.3 pg/mL); a short tetracosactide test confirmed preserved adrenal function (Table 2). Vitamin B12 was reduced at 163 pg/mL (SI: 120 pmol/L) (reference range 196-807 pg/mL [SI: 145-596 pmol/L]), and 25-hydroxy vitamin D was reduced at 8 ng/mL (SI: 20 nmol/L) (reference range > 20 ng/mL [SI: >50 nmol/L]).

**Table 2:** The important endocrine lab results for the patient

Test	Result (conventional units)	Result (SI units)	Reference range
Vitamin B12	163 pg/mL	120 pmol/L	196-807 pg/mL (SI: 145-596 pmol/L)
Vitamin D	8 ng/mL	20 nmol/L	>20 ng/mL (SI: >50 nmol/L)
Adrenocorticotropic hormone (ACTH)	15.7 pg/mL	—	7.2-63.3 pg/mL
Prolactin	101.2 ng/mL	2146 mIU/L	4.0-15.2 ng/mL (SI: 85-323 mIU/L)
Thyroid-stimulating hormone (TSH)	1.65 mIU/L	—	0.45-4.50 mIU/L
Free thyroxine (Free T4)	1.08 ng/dL	13.86 pmol/L	0.70-1.55 ng/dL (SI: 9.0-20.0 pmol/L)
Insulin-like growth factor 1 (IGF-1)	161 ng/mL	161 µg/L	132-348 ng/mL
Total testosterone	270 ng/dL	9.38 nmol/L	300-1077 ng/dL (SI: 10.4-37.4 nmol/L)
Free testosterone	5.5 ng/dL	0.19 nmol/L	5.8-17.9 ng/dL (SI: 0.20-0.62 nmol/L)
Bioavailable testosterone	123 ng/dL	4.27 nmol/L	126-412 ng/dL (SI: 4.36-14.3 nmol/L)
Sex hormone-binding globulin (SHBG)	45.7 nmol/L	45.7 nmol/L	52.8-156.0 nmol/L (SI: 18.3-54.1 nmol/L)
Follicle-stimulating hormone (FSH)	1.4 IU/L	—	1.5-12.4 IU/L
Luteinizing hormone (LH)	7 IU/L	—	1.7-8.6 IU/L
Morning cortisol	3.8 µg/dL	105 nmol/L	5.0-25.0 µg/dL (SI: 138-689 nmol/L)
Stimulated cortisol (baseline)	9.0 µg/dL	248 nmol/L	Peak increase >7.2 µg/dL and peak >14.5 µg/dL
Stimulated cortisol after 30 minutes	20.4 µg/dL	564 nmol/L	—
Stimulated cortisol after 60 minutes	23.3 µg/dL	643 nmol/L	—

Abbreviations: ACTH, adrenocorticotropic hormone; TSH, thyroid-stimulating hormone; Free T4, free thyroxine; IGF-1, insulin-like growth factor 1; SHBG, sex hormone-binding globulin; FSH, follicle-stimulating hormone; LH, luteinizing hormone.

Urine studies showed preserved concentrating ability: spot osmolality 719 to 731 mOsm/kg (SI: 719-731 mOsm/kg) (reference range 150-1150 mOsm/kg) and total 24-hour urine volume 1660 mL (SI: 1.66 L). Urinary sodium excretion was elevated at spot urine sodium 195 mEq/L (SI: 195 mmol/L) (reference range not available; interpreted based on clinical context) and 24-hour urine sodium 269 mEq/day (SI: 269 mmol/day) (reference range 40-220 mEq/day [SI: 40-220 mmol/day]). Lowest urine osmolality at 266 mOsm/kg occurred with serum sodium decline after fluid replacement (Table 3). Other urinary electrolytes were within expected ranges.

**Table 3:** The urine studies results for the patient

Parameter	Result (conventional units)	Result (SI units)	Reference range
24-hour urine volume	1660 mL	1.66 L/day	—
Urine osmolality (spot)	719-731 mOsm/kg	719-731 mOsm/kg	150-1150 mOsm/kg
Urine osmolality (lowest)	266 mOsm/kg	266 mOsm/kg	150-1150 mOsm/kg
24-hour urine sodium	269 mEq/day	269 mmol/day	40-220 mEq/day (SI: 40-220 mmol/day)
Spot urine sodium	up to 195 mEq/L	up to 195 mmol/L	—
Urine creatinine (24 hours)	1.65 g/day	14.56 mmol/day	1.02-2.37 g/day (SI: 9.0-21.0 mmol/day)
Urine urea (24 hours)	2624 mg/day	437 mmol/day	2570-4288 mg/day (SI: 428-714 mmol/day)

Pituitary MRI with contrast was normal, with preserved posterior pituitary bright spot and no masses or stalk abnormalities. Whole-body computed tomography (CT)—which was done to rule out underlying malignancy because of weight loss history—was unremarkable except for a small non-enhancing liver lesion, likely cystic, and simple left renal cysts.

Evaluation of EDS included polysomnography demonstrating a short sleep onset latency (<10 minutes; normal 10-20 minutes), high sleep efficiency (91.6%), reduced rapid eye movement (REM) sleep, and prolonged N2 sleep stage, along with mild obstructive sleep apnea (OSA) (AHI 9.7).

Short sleep onset latency indicates increased sleep propensity, while the high sleep efficiency reflects a consolidated sleep pattern rather than fragmented sleep. The predominance of N2 (light/intermediate sleep stage) sleep with reduced REM suggests altered sleep architecture with relative suppression of deeper sleep stages. Overall, these findings support objectively increased sleep drive with abnormal sleep architecture; although OSA may contribute, the degree of sleepiness appears disproportionate, raising consideration of an underlying central hypersomnia disorder. The polysomnography was repeated due to persistent EDS despite only mild OSA on the initial study and concern for an alternative or additional diagnosis; the discrepancy in AHI (9.7 vs 35.9) may reflect variability in sleep quality and underestimation on the initial study.

Drug-induced sleep endoscopy revealed velopharyngeal, tongue-base, and epiglottic obstruction. In this patient, the diagnosis of NT1 was made clinically based on the presence of cataplexy, hypnagogic hallucinations, and persistent EDS despite treatment of OSA. However, without multiple sleep latency test (MSLT) confirmation, the diagnosis remains presumptive.

## Treatment

Given severe hypernatremia, high serum osmolality, adipsia with preserved renal concentrating ability, and pituitary dysfunction (hypogonadotropic hypogonadism, hyperprolactinemia) with normal MRI, a functional hypothalamic disorder was suspected. The patient was started on scheduled oral fluids and structured feeding, with initial sodium improvement. Testosterone replacement and cabergoline were initiated, with cabergoline prescribed to address hyperprolactinemia and its potential contribution to hypogonadotropic hypogonadism, despite the absence of a visible pituitary lesion, consistent with a hypothalamic (“stalk effect”) mechanism. Noncompliance led to recurrent hypernatremia, later stabilized with adherence.

The patient was also treated with continuous positive airway pressure (CPAP). For the suspected narcolepsy, he was started on modafinil 100 mg twice daily and methylphenidate 10 mg three times daily which significantly improved daytime sleepiness and fatigue.

## Outcome and follow-up

On follow-up, the patient is not fully adherent to CPAP, but while on medications, his sleep pattern has normalized. He continues to struggle with structured fluid intake and meals and is actively adopting lifestyle routines and schedules to manage his symptoms. His serum sodium remains elevated, with the latest reading reaching 160 mEq/L (SI: 160 mmol/L), and he is being closely monitored in the outpatient setting.

## Discussion

Plasma osmolality is tightly regulated by hypothalamic osmoreceptors, thirst perception, and AVP secretion and action. Even small osmotic increases normally trigger thirst and AVP release. Chronic hypernatremia in an alert patient with access to water is unusual, suggesting hypothalamic dysfunction [5].

Hypothalamic dysfunction may arise from a broad range of etiologies, including structural lesions (as tumors, trauma, or infiltrative disorders), inflammatory or autoimmune processes, congenital abnormalities, and functional disturbances without overt radiological findings [2, 6, 7]. In many reported cases of adipsic hypernatremia, no structural cause is identified, suggesting selective or microscopic hypothalamic injury affecting osmoregulatory pathways [2, 6]. In our patient, the absence of structural abnormalities on MRI and lack of prior neurological insult make a functional etiology more likely. Notably, severe OSA may contribute to hypothalamic dysfunction through chronic intermittent hypoxia, oxidative stress, and neuroinflammation, potentially impairing osmoreceptor signaling and broader neuroendocrine regulation [4]. While an autoimmune mechanism remains a theoretical consideration, the available data in this case do not support a definitive underlying cause, and the presentation is best interpreted as functional hypothalamic dysregulation [8].

Damage to osmoreceptive hypothalamic regions can uncouple thirst from AVP-mediated renal water conservation, causing hypernatremia despite preserved urine concentration [1, 5]. Adipsic or hypodipsic hypernatremia represents a rare but well-characterized manifestation of hypothalamic dysfunction. Early descriptions highlighted patients with absent thirst responses, chronic hypernatremia, and variable AVP secretion, often without polyuria or overt dehydration. Subsequent reports refined this concept, demonstrating that AVP secretion may remain partially or fully preserved through non-osmotic pathways, leading to concentrated urine despite marked hyperosmolality [5, 6].

The lack of thirst despite ongoing vasopressin activity distinguishes adipsic hypernatremia from classic central diabetes insipidus. Because urine can still be concentrated, this may falsely reassure clinicians and delay recognition of the underlying hypothalamic problem [5, 7]. Crowley and colleagues, in one of the largest adult case series (n = 13) of adipsic diabetes insipidus, emphasized that failure to recognize the pattern of adipsia with disordered osmoregulation of thirst and vasopressin can delay diagnosis and expose patients to recurrent, potentially life-threatening hypernatremic complications [2].

In contrast, adipsic diabetes insipidus reflects a more advanced disruption of water homeostasis, in which loss of thirst perception is accompanied by impaired vasopressin secretion and overt polyuria, often requiring desmopressin in addition to fixed fluid intake. This differs from our patient's presentation, where absent thirst occurred in the setting of preserved urine concentration, supporting a diagnosis of adipsic hypernatremia rather than adipsic diabetes insipidus [9, 10].

Hypothalamic dysfunction frequently involves multiple systems, including appetite, sleep-wake cycles, and pituitary hormone regulation. While hypothalamic dysfunction is more commonly associated with hyperphagia and weight gain, weight loss has also been described in selected cases, particularly when associated with reduced appetite, behavioral changes, or coexisting systemic factors. In our patient, extensive evaluation did not identify gastrointestinal, malignant, or chronic infectious causes. The weight loss may reflect reduced oral intake related to hypothalamic dysregulation of appetite and thirst.

In this case, hyperprolactinemia likely reflects impaired hypothalamic dopaminergic inhibition, contributing to suppression of the gonadal axis, with cabergoline initiated to address this effect. The pattern of low FSH and testosterone with preserved LH suggests partial hypothalamic–pituitary–gonadal dysregulation rather than complete central failure. Overall, this endocrine profile reinforces the interpretation of adipsic hypernatremia as part of a broader hypothalamic syndrome rather than an isolated disorder of water balance [2].

The absence of abnormalities on conventional MRI, as observed in our patient, does not exclude clinically significant hypothalamic disease. Several reports document normal structural imaging despite clear biochemical and clinical evidence of hypothalamic dysfunction, suggesting involvement at a microscopic or network level beyond the resolution of routine imaging modalities [2, 6]. Recent literature further emphasizes that adipsic states represent a distinct and high-risk variant within the spectrum of hypothalamic and central diabetes insipidus disorders, reinforcing the need for clinical recognition despite preserved urine concentration and normal imaging [7, 10].

In parallel, emerging literature has proposed autoimmune-mediated hypothalamic dysfunction as a potential mechanism in selected cases, supported by the identification of autoantibodies targeting osmoregulatory or sodium-sensing pathways in a small subset of patients [8]. Autoimmune antibody testing was not performed; therefore, autoimmune hypothalamic pathology could not be evaluated in this case.

Narcolepsy and OSA frequently coexist, with OSA reported in 24.8% to 51.4% of adults with NT1, and this overlap may delay diagnosis and appropriate management. In our patient, polysomnography and drug-induced sleep endoscopy confirmed severe OSA, while narcolepsy with cataplexy was diagnosed clinically based on cataplexy, hypnagogic hallucinations, and persistent EDS despite CPAP. This overlap is thought to involve orexin (hypocretin) dysfunction, with bidirectional interactions whereby orexin deficiency may worsen ventilatory stability and OSA-related intermittent hypoxia may further impair orexin pathways, potentially masking coexisting narcolepsy and complicating interpretation of sleep testing such as MSLT [11]. Continuous positive airway pressure alone often fails to resolve EDS in such patients, and wake-promoting agents including modafinil, methylphenidate, pitolisant, and solriamfetol may be used as adjunct therapy alongside positive airway pressure [3, 12].

Obstructive sleep apnea disrupts hypothalamic function through intermittent hypoxia, sleep fragmentation, and chronic stress, leading to dysregulation of the hypothalamic–pituitary–adrenal axis and impaired neuroendocrine control [4]. Recurrent hypoxemia-reoxygenation induces oxidative stress, sympathetic activation, and hypertension via ischemic and hypoxia-inducible factors pathways [13]. It also alters hypothalamic signaling in the paraventricular nucleus and increases systemic inflammation mediated by nuclear factor-kappa B. Furthermore, sleep fragmentation elevates cortisol levels and alters ACTH dynamics, which is partially reversible with CPAP [3, 11, 14, 15]. Together, these mechanisms link OSA to functional hypothalamic and neuroendocrine dysfunction.

This case raises the possibility that severe OSA may contribute to functional hypothalamic dysfunction; however, this association remains speculative and cannot be established from a single case. In our patient, adipsic hypernatremia, preserved renal concentrating ability, intermittent hypogonadotropic hypogonadism, hyperprolactinemia, and sleep-wake disorders, despite normal hypothalamic-pituitary imaging, suggest a syndromic functional hypothalamic dysfunction. Obstructive sleep apnea–related injury, via intermittent hypoxia and neuroinflammation, may disrupt thirst and neuroendocrine regulation, unifying these abnormalities.

## Learning points

- Chronic hypernatremia with absent thirst suggests hypothalamic dysfunction, even with normal urine concentration.
- Adipsic hypernatremia may occur despite normal brain and hypothalamic-pituitary neuroimaging, indicating functional hypothalamic impairment.
- Endocrine and sleep disturbances often coexist, reflecting the syndromic nature of hypothalamic dysfunction and highlighting the need for multidisciplinary management.
- Obstructive sleep apnea and narcolepsy can exacerbate hypothalamic dysregulation and water balance disorders.

## Contributors

M.D. contributed to patient care, acquisition and interpretation of clinical data, drafting and critical revision of the manuscript, and final approval of the submitted version. M.A. contributed to patient care, interpretation of clinical data, critical revision of the manuscript for important intellectual content, and final approval of the submitted version. M.O.M. contributed to patient care, interpretation of clinical data, critical revision of the manuscript for important intellectual content, and final approval of the submitted version. A.A. contributed to patient care, interpretation of clinical data, critical revision of the manuscript for important intellectual content, and final approval of the submitted version. M.N. contributed to patient care, interpretation of clinical data, critical revision of the manuscript for important intellectual content, and final approval of the submitted version.

## Data Availability

Restrictions apply to the availability of some or all data generated or analyzed during this study to preserve patient confidentiality or because they were used under license. The corresponding author will on request detail the restrictions and any conditions under which access to some data may be provided.
